# Machine learning for prognostic impact in elderly unresectable hepatocellular carcinoma undergoing radiotherapy

**DOI:** 10.3389/fonc.2025.1585125

**Published:** 2025-04-16

**Authors:** Yuhui Shi, Xianguo Liu

**Affiliations:** Department of Oncology, 363 Hospital, Chengdu, China

**Keywords:** hepatocellular carcinoma, radiotherapy, survival prognosis, machine learning, survival

## Abstract

**Background/Aim:**

This study develops a machine learning-based predictive model to evaluate the survival outcomes of elderly patients with unresectable hepatocellular carcinoma (HCC) undergoing radiotherapy.

**Methods:**

The 2377 patients from SEER database were divided into training and internal validation cohorts. Additionally, 99 patients from our hospital were used for an external validation cohort. In the training cohort, 101 machine learning-based radiomics models were developed, and the optimal model’s performance was subsequently evaluated in both the internal and external validation cohorts.

**Results:**

The StepCox + GBM model demonstrated the highest C-index of 0.7 in the training cohort. The model was further evaluated using area under the receiver operating characteristic (AUC-ROC) curves, with AUC values ranging from 0.736 to 0.783, indicating strong predictive performance. Furthermore, the calibration curve and decision curves confirmed that the model had good predictive performance.

**Conclusions:**

The StepCox + GBM model could help optimize the use of radiotherapy for elderly HCC patients, improving survival outcomes and guiding personalized treatment strategies.

## Introduction

Hepatocellular carcinoma (HCC) is one of the most prevalent cancers globally, with high incidence and mortality rates, particularly in regions with a high prevalence of hepatitis B virus (HBV) and hepatitis C virus (HCV) (1, 2). While surgical resection remains the preferred treatment for localized HCC, a significant number of patients are diagnosed at an advanced stage, where surgery is not feasible. For these patients, treatment options include transarterial chemoembolization (TACE), systemic therapy, and radiotherapy (3–5). Radiotherapy, especially for unresectable tumors, offers a non-invasive alternative to relieve symptoms, control tumor growth, and improve quality of life (6).

The prognosis for elderly HCC patients is relatively poor due to both their advanced age and the challenges associated with managing comorbidities (7). Elderly patients often present with more advanced stages of HCC and are less likely to tolerate aggressive treatments such as surgery or systemic therapies (8, 9). Radiotherapy has emerged as a promising modality for improving survival outcomes in elderly patients with unresectable HCC (10). However, there is a lack of reliable predictive models that can accurately identify elderly patients who are most likely to benefit from radiotherapy, thereby limiting the ability to tailor treatment strategies effectively.

Machine learning (ML) offers a powerful approach to constructing predictive models from complex datasets (11). ML algorithms can identify patterns and relationships within large datasets that may not be immediately apparent through traditional statistical methods (12). By leveraging the capabilities of machine learning, researchers have the potential to develop more precise and individualized treatment strategies for elderly patients with unresectable HCC (13–15). These models could help identify high-risk patients, thus assisting clinicians in making more informed treatment decisions.

To develop a robust predictive model for elderly patients with unresectable HCC undergoing radiotherapy, we will integrate data from the SEER database with data from our hospital. Through the application of machine learning techniques, we aim to construct a model that predicts the treatment outcomes for elderly patients with unresectable HCC undergoing radiotherapy, identifies high-risk patients, and assists clinicians in making more informed treatment decisions.

## Materials and methods

### Eligibility criteria

This study included 2377 unresectable HCC patients who received beam radiation from the SEER-17 registries (2000–2020) (**Supplementary Figure S1**), and 99 unresectable HCC patients treated with Gamma knife radiosurgery (GKR) across 363 hospitals (16). The inclusion criteria were: (a) no surgical treatment recommended, (b) received external beam radiotherapy, (c) complete information available, and (d) age ≥ 60 years. Patients with incomplete follow-up data or those who underwent surgery were excluded. The study was approved by the institutional review boards.

### GKR

GKR was executed using a Treatment Planning System (TPS), where radiation oncologists delineated the treatment area based on contrast-enhanced CT images. The gross target volume (GTV), including the primary liver tumor, was defined using these imaging techniques, and a 5–10 mm margin was added around the GTV to create the planning target volume (PTV) via TPS. The median tumor margin dose was 42 Gy (ranging from 39 to 42 Gy).

### Machine learning models

The 2377 patients were randomly divided into a training cohort (n = 1661) and an internal validation cohort (n = 716) at a 7:3 ratio. Additionally, data from our hospital were used for an external validation cohort (n = 99). Univariate Cox regression analysis was used to identify clinical factors affecting overall survival (OS) in the training cohort. Using these factors, 101 machine learning-based radiomics models were developed. The model’s performance was then assessed in both the internal and external validation cohorts using Harrell’s C-index, decision curve analysis (DCA), calibration curves, and receiver operating characteristic (ROC) curves.

### Statistical analysis

Categorical variables between the training, internal validation, and external validation cohorts were compared using the chi-square test. All statistical analyses were conducted using R software (version 3.3.2). A p-value of < 0.05 was regarded as statistically significant.

## Results

### Clinical features

In the training cohort, the majority of patients were male (78.5%), White (74.9%), and had stage T1 tumors (37.8%). The internal validation cohort also had a high proportion of male patients (77.0%) and White patients (76.4%), with T1 tumors being the most common (34.2%). The external validation cohort consisted entirely of patients from the “Other” race group (100%) and had a higher proportion of T2 tumors (31.3%). In terms of AFP status, the majority of patients in the training and internal validation cohorts were AFP positive (45.4% and 42.0%, respectively), while the external validation cohort had an even split between positive and negative AFP. The three patient groups showed differences in race, grade, T, N, M, tumor stage, AFP levels, and tumor size (**Table 1**).

**Table 1:** Clinical characteristics of HCC patients.

	Training cohort	Internal validation	External validation	p
Patient	1661	716	99	
Sex				0.645
Female	357 (21.5)	165 (23.0)	20 (20.2)	
Male	1304 (78.5)	551 (77.0)	79 (79.8)	
Race				<0.001
White	1244 (74.9)	547 (76.4)	0	
Black	185 (11.1)	68 (9.5)	0	
Other	232 (14.0)	101 (14.1)	99 (100.0)	
Grade				<0.001
1	172 (10.4)	82 (11.5)	0	
2	181 (10.9)	76 (10.6)	0	
3	112 (6.7)	58 (8.1)	0	
4	8 (0.5)	5 (0.7)	0	
Unknown	1188 (71.5)	495 (69.1)	99 (100.0)	
T				
T1	628 (37.8)	245 (34.2)	29 (29.3)	<0.001
T2	251 (15.1)	106 (14.8)	31 (31.3)	
T3	329 (19.8)	162 (22.6)	14 (14.1)	
T4	116 (7.0)	52 (7.3)	25 (25.3)	
Tx	337 (20.3)	151 (21.1)	0	
N				
N0	1189 (71.6)	509 (71.1)	72 (72.7)	<0.001
N1	152 (9.2)	71 (9.9)	27 (27.3)	
Unknown	320 (19.3)	136 (19.0)	0	
M				
M0	800 (48.2)	336 (46.9)	79 (79.8)	<0.001
M1	739 (44.5)	330 (46.1)	20 (20.2)	
Unknown	122 (7.3)	50 (7.0)	0	
Stage				<0.001
I	412 (24.8)	158 (22.1)	4 (4.0)	
II	137 (8.2)	62 (8.7)	23 (23.2)	
III	162 (9.8)	67 (9.4)	29 (29.3)	
IV	765 (46.1)	347 (48.5)	43 (43.4)	
Unknown	185 (11.1)	82 (11.5)	0	
AFP				<0.001
Negative	269 (16.2)	129 (18.0)	49 (49.5)	
Positive	754 (45.4)	301 (42.0)	50 (50.5)	
Unknown	638 (38.4)	286 (39.9)	0	
Size, cm				
< 5	643 (38.7)	271 (37.8)	64 (64.6)	<0.001
≥ 5	704 (42.4)	311 (43.4)	35 (35.4)	
Unknown	314 (18.9)	134 (18.7)	0	

HCC, hepatocellular carcinoma; AFP, alpha fetoprotein.

### Machine learning models construction

**Figure 1:**
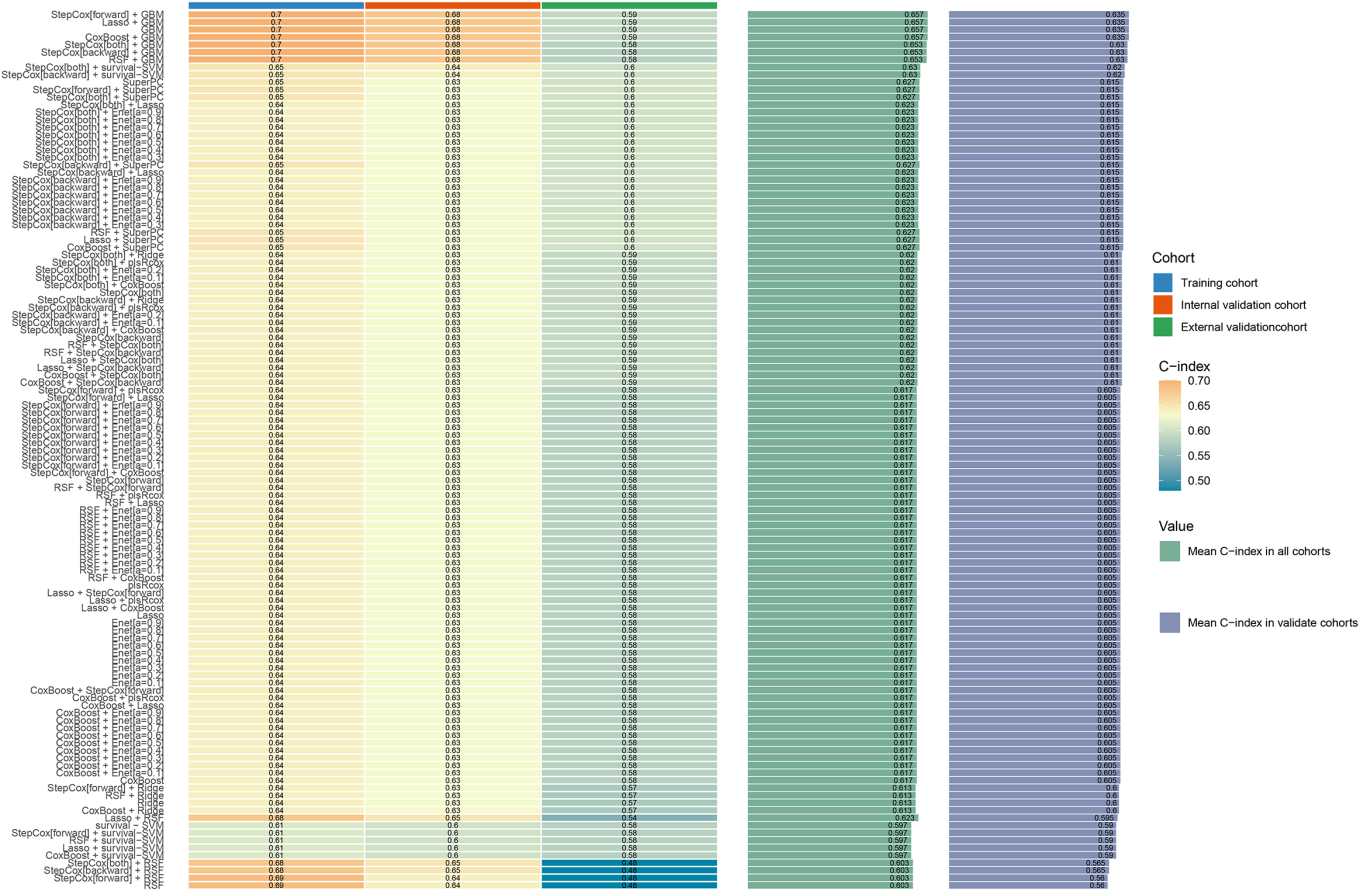
Compare the C-index of 101 machine learning algorithms in the training set, internal validation set, and external validation set.

**Figure 2:**
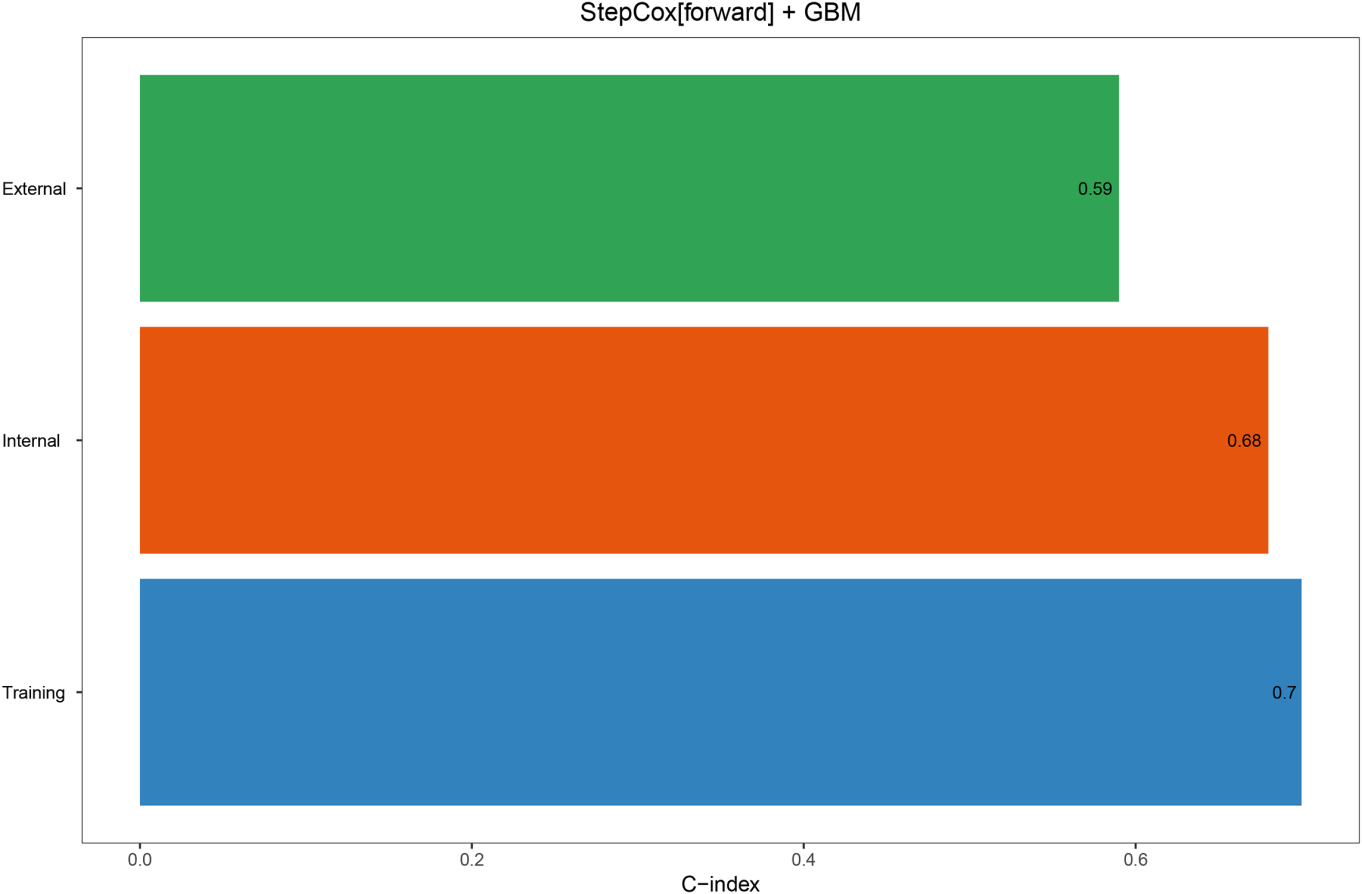
The C-index of the StepCox + GBM model in the training set, internal validation set, and external validation set.

**Figure 3:**
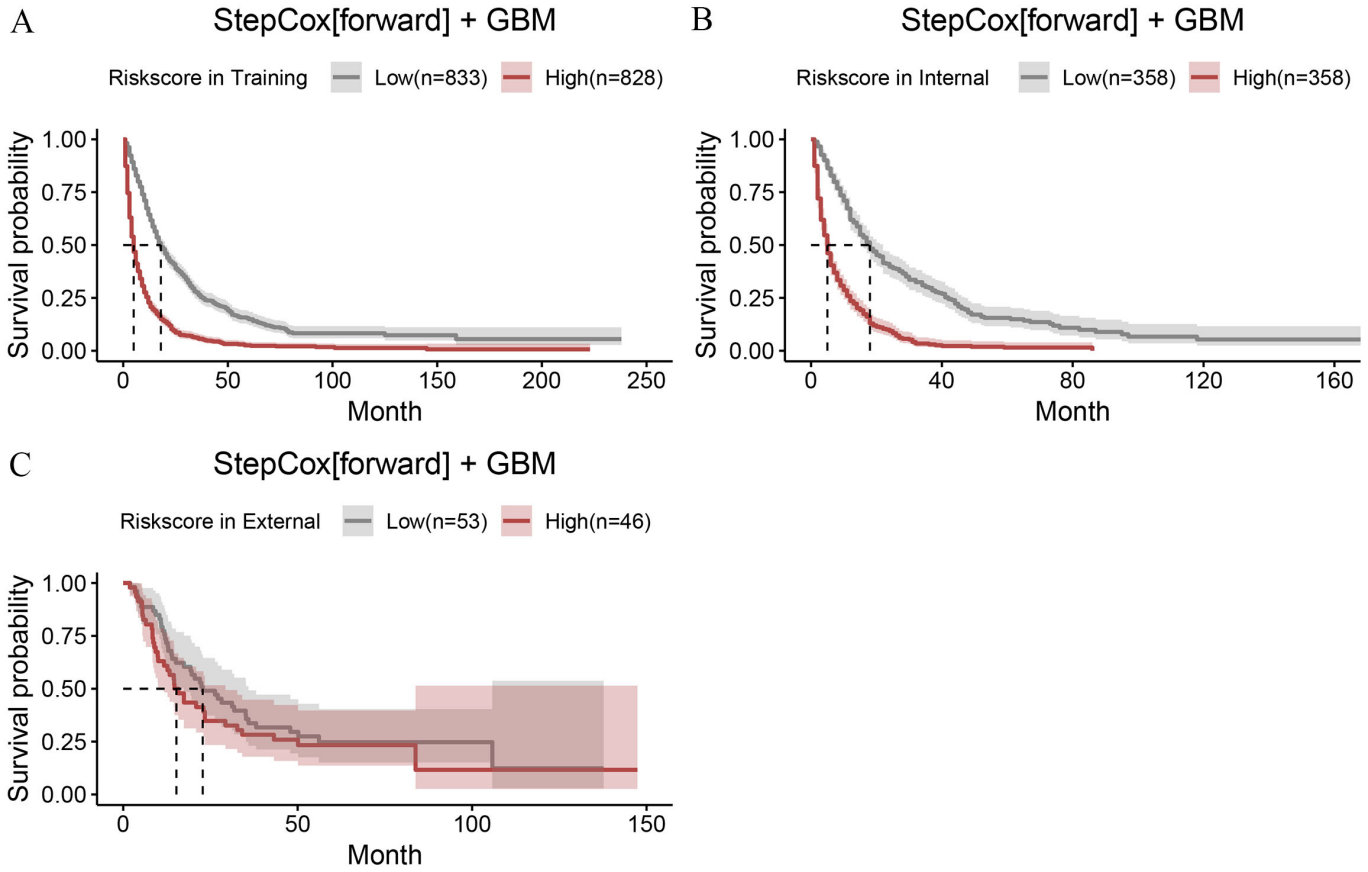
The risk scores calculated using the StepCox + GBM model were divided into two groups (high and low) based on the median cutoff value. The OS of the two groups was compared in the training set **(A)**, internal validation set **(B)**, and external validation set **(C)**.

In the univariate COX analysis, Sex, Grade, T, N, M, Stage, and Size were confirmed as prognostic indicators for OS. Subsequently, 101 machine learning models were constructed in the training set, and StepCox[forward] + GBM exhibited the highest concordance index (C-index) of 0.7 (**Figure 1**). In the internal validation set and external validation set, the C-indices were 0.68 and 0.59, respectively (**Figure 2**). Based on the StepCox[forward] + GBM model, the risk score for each patient was calculated and divided into high-risk and low-risk groups based on the median. The high-risk group showed a shorter OS compared to the low-risk group in both the training set (**Figure 3A**) and the internal (**Figure 3B**) and external validation sets (**Figure 3C**).

### Model evaluation

**Figure 4:**
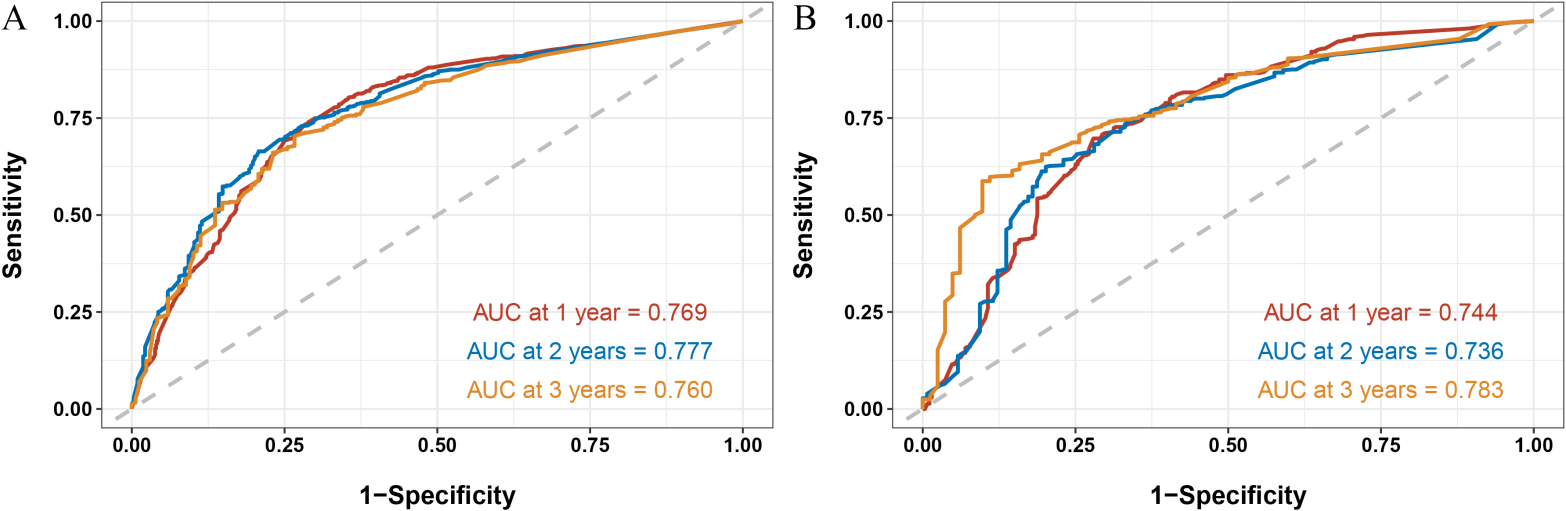
In the internal validation set **(A)** and external validation set **(B)**, the StepCox + GBM model was used to evaluate the Area Under the Curve - Receiver Operating Characteristic for 1-, 2-, and 3- year overall survival.

**Figure 5:**
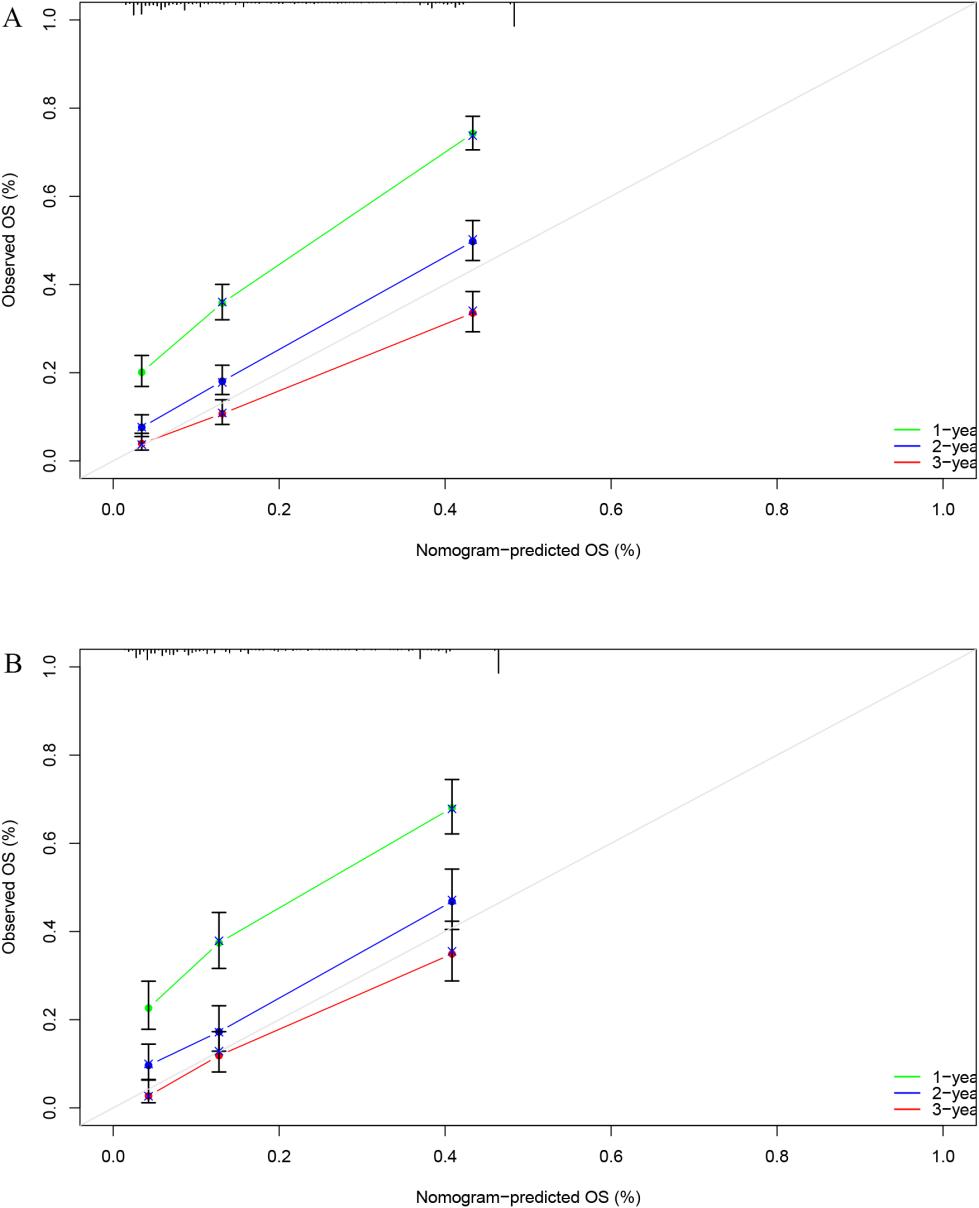
In the internal validation set **(A)** and external validation set **(B)**, the calibration curve confirmed that the StepCox + GBM model has good predictive performance.

**Figure 6:**
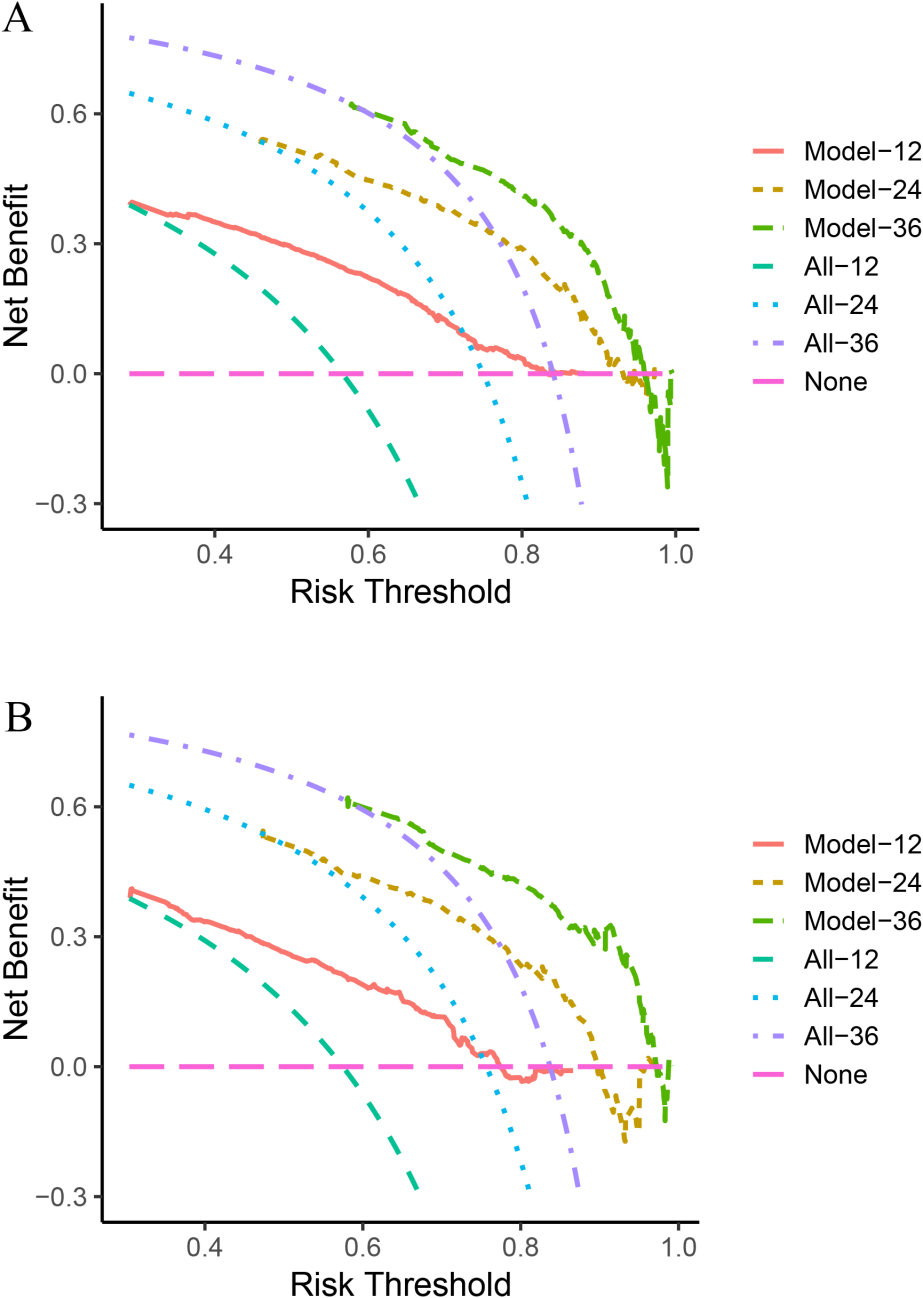
In the internal validation set **(A)** and external validation set **(B)**, the decision curve analysis confirmed that the StepCox + GBM model has good predictive performance.

Next, we calculated the Area Under the ROC (AUC-ROC) values for the 1, 2, and 3-year OS based on the risk score. In the internal validation set, the AUC values were 0.769, 0.777, and 0.760, respectively (**Figure 4A**), and in the external validation set, the values were 0.744, 0.736, and 0.783, respectively (**Figure 4B**). Furthermore, the calibration curves (**Figures 5A, B**) and decision curves (**Figures 6A, B**) confirmed that the model had good predictive performance.

## Discussion

HCC is a major health burden worldwide, with high mortality and incidence rates. The prognosis for patients with unresectable HCC is generally poor, and the treatment options are limited (17). This study is clinically significant as it aims to address a critical gap in personalized treatment for elderly patients with unresectable HCC. By leveraging data from both the SEER database and our hospital, this research builds a predictive model to help clinicians make more informed treatment decisions. By predicting the outcomes of radiotherapy for these patients, the model may guide the selection of appropriate treatments, ensuring that high-risk patients are identified and provided with the necessary care. The ability to predict treatment outcomes and tailor interventions accordingly could potentially improve OS and quality of life in elderly HCC patients, who are often more vulnerable due to the challenges posed by comorbidities and the advanced stage of their disease (18).

The treatment of elderly patients with unresectable HCC remains one of the most challenging aspects of managing liver cancer (19). These patients often present with more advanced stages of the disease, complicating treatment strategies (20). Additionally, their advanced age, frailty, and multiple comorbidities reduce their ability to tolerate aggressive treatments such as surgical resection or systemic therapies like chemotherapy (21). As a result, treatment options are limited, and survival outcomes for elderly patients are typically poor. In this context, radiotherapy has emerged as an essential non-invasive treatment option for elderly patients with unresectable tumors. Radiotherapy can help control tumor growth, alleviate symptoms such as pain or bleeding, and improve the quality of life. It is particularly valuable for those who are not candidates for surgery or other invasive treatments (22, 23). Despite its benefits, the selection of patients who will most benefit from radiotherapy remains a clinical challenge. Therefore, predictive models, such as the one presented in this study, could greatly aid in the identification of patients who are most likely to respond to radiotherapy, optimizing treatment strategies.

The machine learning-based predictive model developed in this study demonstrates strong predictive performance. The model incorporates clinical factors identified through univariate Cox regression analysis, such as sex, grade, T stage, N stage, M stage, tumor size, and others, to predict overall survival outcomes for elderly patients with unresectable HCC undergoing radiotherapy. The model’s performance was validated using both internal and external validation cohorts, showing a concordance index (C-index) of 0.7 in the training cohort, 0.68 in the internal validation cohort, and 0.59 in the external validation cohort, indicating a good predictive capability. Furthermore, the AUC-ROC values for 1, 2, and 3-year OS, ranging from 0.736 to 0.783 in both the internal and external validation sets, further underscore the model’s robustness. By calculating individual risk scores and categorizing patients into high-risk and low-risk groups, clinicians can better tailor their treatment strategies for elderly HCC patients. This risk stratification could potentially guide the use of radiotherapy, ensuring that high-risk patients receive appropriate interventions while avoiding unnecessary treatments for low-risk patients.

Recent studies on radiotherapy for HCC have highlighted several promising developments. Advances in techniques such as stereotactic body radiotherapy (SBRT) and proton therapy have shown significant improvements in treatment efficacy, especially for patients with unresectable tumors (24, 25). SBRT, with its precision in targeting tumors while sparing surrounding healthy tissues, has demonstrated high rates of tumor control and promising survival outcomes in both early and advanced stages of HCC (26, 27). These innovations in radiotherapy techniques and their integration with machine learning models make this study particularly relevant in the evolving landscape of liver cancer treatment.

We evaluated 101 different machine learning algorithms and identified the StepCox + GBM model as the one with the best predictive performance. This combined model demonstrates superior predictive performance compared to single-model approaches, offering significant clinical value, especially in the context of personalized medicine (28). By integrating these two powerful algorithms, the study leverages their complementary strengths—StepCox’s ability to identify significant clinical factors and GBM’s capability to handle complex non-linear relationships in the data (29, 30). This synergy enhances the model’s overall accuracy and robustness, providing a more reliable tool for predicting survival outcomes in elderly patients with unresectable HCC undergoing radiotherapy. The ability to identify high-risk patients and predict their treatment response before therapy begins would help clinicians optimize resources and improve patient outcomes. Moreover, the combined model has the potential to continuously evolve as more data becomes available, further refining its accuracy and predictive power, making it a valuable asset for clinical decision-making and individualized treatment plans (31).

The predictive model offers several clinical benefits: it enables personalized treatment decisions by identifying high-risk patients who may benefit from more aggressive or additional therapies, while allowing low-risk patients to avoid overtreatment and its associated toxicities. Moreover, by tailoring treatment based on predicted outcomes, the model facilitates optimized resource allocation, ensuring that the patients most likely to benefit receive appropriate interventions. Finally, by providing an objective, data-driven estimation of survival outcomes, the model enhances prognostic accuracy, thereby supporting clinical decision-making and potentially improving overall survival and quality of life for this vulnerable patient population.

Despite the promising results, there are several limitations to this study. First, the model was based on retrospective data from the SEER database and our hospital, which may introduce selection bias. The external validation cohort, while adding strength to the study, may still differ in terms of patient demographics and treatment protocols, potentially affecting the model’s generalizability. Second, the model does not account for potential changes in treatment regimens or advancements in radiotherapy techniques that may have occurred after the data collection period. Additionally, the model’s reliance on clinical factors such as tumor stage and size may not fully capture the complexity of individual patients’ responses to radiotherapy. Future studies could incorporate genetic, molecular, and radiomic data to further refine the model and improve its predictive accuracy.

## Conclusion

This study presents a machine learning-based predictive model that shows strong performance in predicting the survival outcomes of elderly patients with unresectable HCC undergoing radiotherapy. The model’s ability to stratify patients into high-risk and low-risk groups based on clinical factors provides valuable insights that can aid in clinical decision-making.

## Data Availability

The original contributions presented in the study are included in the article/**Supplementary Material**. Further inquiries can be directed to the corresponding author.
