# Supplementary figures and images for: Machine learning for prognostic impact in elderly unresectable hepatocellular carcinoma undergoing radiotherapy
Source: Front Oncol. 2025 Apr 16;15:1585125. doi: 10.3389/fonc.2025.1585125 (PMC12040856; doi:10.3389/fonc.2025.1585125)

**Supplementary Fig. 1.** Patient selection.

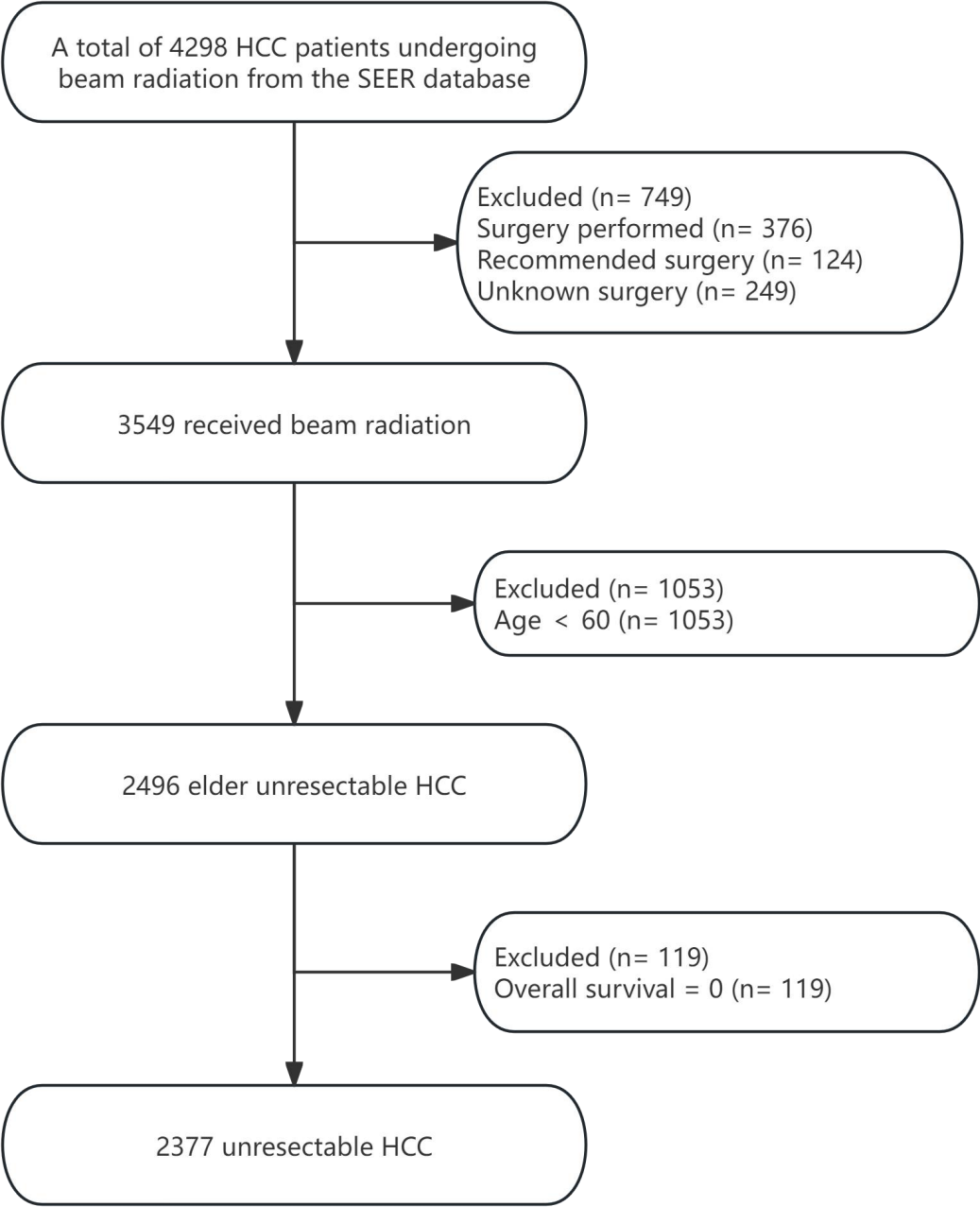

Supplement: Supplementary file 1 [file DataSheet1.pdf]
